# Risk Factors for COVID-19 in a Retired FDNY WTC-Exposed Cohort

**DOI:** 10.3390/ijerph19158891

**Published:** 2022-07-22

**Authors:** Krystal L. Cleven, Rachel Zeig-Owens, David G. Goldfarb, Theresa Schwartz, David J. Prezant

**Affiliations:** 1 Pulmonary Medicine Division, Department of Medicine, Montefiore Medical Center and Albert Einstein College of Medicine, Bronx, NY 10467, USA; rachel.zeig-owens@fdny.nyc.gov (R.Z.-O.); david.goldfarb@fdny.nyc.gov (D.G.G.); theresa.schwartz@fdny.nyc.gov (T.S.); david.prezant@fdny.nyc.gov (D.J.P.); 2 The Bureau of Health Services and the FDNY World Trade Center Health Program, Fire Department of the City of New York, Brooklyn, NY 11201, USA; 3 Division of Epidemiology, Department of Epidemiology and Population Health, Albert Einstein College of Medicine, Bronx, NY 10461, USA

**Keywords:** World Trade Center, COVID-19, FDNY, Fire Department of City of New York (FDNY), occupational lung disease

## Abstract

We evaluated the incidence and risk factors for COVID-19 in a prospectively followed cohort of Fire Department of the City of New York (FDNY) World Trade Center (WTC)-exposed workers, thus reducing the potential for selection bias, a limitation in published studies of hospitalized individuals. Participants were retired FDNY WTC-exposed rescue/recovery workers with ≥1 medical visit between 1 March 2020 and 1 August 2021. The cumulative incidence was calculated using self-reported COVID-19 diagnoses. Cox regression was performed to evaluate the association of WTC-exposure and COVID-19, adjusting for history of comorbidities, age, race, work assignment (emergency medical service providers vs. firefighter), and sex. The cumulative incidence of COVID-19 was 130 per 1000. The adjusted models showed the risk of infection was greater in those with highest WTC exposure versus less exposure (hazard ratio (HR) = 1.14 (95% CI 1.00–1.31)). Older age was associated with a lower risk of infection HR = 0.97 (95% CI 0.96–0.98). WTC-associated diseases (obstructive airways disease and interstitial lung disease) were not COVID-19 risk factors. This study is the first to show an association between WTC exposure and the risk of COVID-19. While participants are retired from FDNY work, the youngest individuals may still be in the workforce, explaining why younger age was a significant risk for COVID-19.

## 1. Introduction

The first case of coronavirus disease 2019 (COVID-19) was identified in New York State on 1 March 2020, and soon after New York City became the epicenter of the pandemic. As of 20 May 2022, over 82 million COVID-19 cases and over one million deaths have occurred in the USA, with just over 5.3 million cases and over 68,000 deaths occurring in New York State (NYS) [1]. This study evaluated the incidence of and potential risk factors for COVID-19 in the retired Fire Department of the City of New York (FDNY) World Trade Center (WTC)-exposed cohort. The cohort includes over 10,000 participants who are retired and have a history of working at the WTC site as an FDNY firefighter or emergency medical services (EMS) worker on or soon after 11 September 2001 (9/11). This cohort has undergone annual health assessments beginning soon after 9/11, which include physical and mental health questionnaires, physical exams, evaluation of health characteristics (e.g., weight, height, and smoking status), laboratory tests, spirometry, chest radiography, self-reported and physician diagnosed health conditions (including conditions related to WTC-exposure) [2]. Since this cohort has been prospectively followed for nearly two decades, the potential for misclassification of risk factors and selection bias is reduced when compared to other cross-sectional and short-term cohort studies, which primarily include hospitalized patients [3,4,5]. Additionally, we have previously showed that in the FDNY WTC-exposed cohort, rescue/recovery work at the disaster site was associated with obstructive pulmonary diseases that are also risk factors for severe COVID-19. Further, Morozova and colleagues evaluated the cumulative incidence of COVID-19 in a cohort of WTC-exposed workers and volunteers residing in Long Island, New York, who were prospectively followed soon after 9/11 and found a cumulative incidence of approximately 22% through August 2020 using positive antibody testing in the electronic medical records (EMR) or self-reported positive polymerase chain reaction test [6]. The incidence rate was higher than the seroprevalence of the general population in the USA (<10%) and New York State (14%), but it was similar to the hardest hit area in the New York City metro area (6.9–22.7%), over a similar time period [6,7,8,9]. This suggests that the occupational exposure that occurred on or after 9/11 is a unique risk factor for COVID-19 and/or more severe disease. Since our retired FDNY cohort is followed prospectively, the goals of this study included quantifying the cumulative incidence of COVID-19 from the start of the pandemic and to determine if WTC exposure, as well as other health conditions, were associated with COVID-19.

## 2. Materials and Methods

### 2.1. Study Population

The source population for this observational cohort study consists of 10,245 FDNY WTC firefighters and EMS workers who were part of the FDNY-WTC Health Program, who consented to research, and were alive and retired from FDNY work on 1 March 2020. The FDNY-WTC Health Program was created shortly after 9/11 to provide comprehensive physical and mental health services to all active and retired FDNY members who responded to the 9/11 attacks. Annual monitoring exams have included a physical exam, laboratory data, chest X-ray, spirometry, and self-administered physical and mental health questionnaires since 2001. Since 2020, questionnaires included information about self-reported COVID-19 diagnoses. Since our study evaluated risk factors for COVID-19, those without at least two years of health monitoring data prior to 1 March 2020 were excluded. Additionally, to calculate the cumulative incidence rate of self-reported COVID-19, we excluded anyone who did not have a followup exam between 1 March 2020 and the end of the study (1 August 2021). The final study sample included 8256 retired FDNY rescue/recovery workers. The Montefiore Medical Center/Albert Einstein College of Medicine Institutional Review Board approved this study (#07-09-320).

### 2.2. Demographics, WTC Exposure History, and Clinical Characteristics

Demographics include age on 1 March 2020, sex, work assignment while actively employed (EMS and firefighter), and self-reported race. Clinical characteristics included self-reported smoking status (ever and never), height, weight (most recent), self-reported or physician-recorded hypertension diagnosis at any time before 1 March 2020, WTC-associated obstructive airways disease (OAD) (asthma, chronic obstructive pulmonary disease [COPD], chronic bronchitis, and emphysema), as well as WTC-associated interstitial lung disease (ILD) (sarcoidosis or pulmonary fibrosis). Body Mass Index (BMI) was calculated by dividing weight (kg) by height squared (m^2^), and obesity was defined as being ≥30 kg/m^2^. OAD and ILD diagnoses were retrieved from the FDNY EMR. The number of healthcare interactions with the WTC Health Program were obtained from the medical records. Vaccination status and date were obtained from the FDNY Bureau of Health Services vaccination records. A participant was considered to be vaccinated for COVID-19 if they received at least one vaccine dose prior to the end of the study period. The degree of WTC exposure has been characterized previously by time of first arrival to the WTC site with those who arrived on 9/11 as the most exposed to the WTC dust cloud (i.e., fine particulate matter) and those who arrived after or on 12 September 2001 as less exposed [10,11]. For this study, a participant’s exposure was dichotomized as higher exposed (arrived on 9/11) or lower exposed (on or after 12 September 2001).

### 2.3. COVID-19 Case Definition

A diagnosis of COVID-19 was identified as positive if participants self-reported COVID-19, a COVID-19 related hospitalization, or died from COVID-19 as reported to the WTC Health Program at any time between 1 March 2020 and 1 August 2021. Severe COVID-19 was defined as either requiring hospitalization or causing death.

### 2.4. Statistical Analyses

Descriptive statistics of the study sample were assessed as proportions, medians and interquartile ranges, or means and standard deviations (SDs), as appropriate. The cumulative incidence of COVID-19 was calculated by reported disease divided by the number of participants at the start of study (1 March 2020). However, it is possible that those with more severe comorbidities or a history of higher WTC exposure utilize the healthcare provided by the FDNY more, thus, giving them more opportunities to report COVID-19 infection. To correct for this potential bias, the incidence rate was also calculated, and person-time was calculated as the time between 1 March 2020 and either the day the participant informed the FDNY that they had COVID-19 or the last day they interacted with the WTC health program during the study period, as this was their last opportunity to report COVID-19. Incidence rate comparisons were made for interested variables (WTC exposure—high vs. low; work assignment—firefighter vs. EMS history). Additionally, the number of healthcare interactions with the WTC Health Program were obtained from the medical records and assessed descriptively by calculating median/interquartile range. Risk factors for COVID-19 were evaluated using Cox proportional hazard regression to evaluate hazard ratios (HR) with 95% confidence intervals (CI) for self-reported COVID-19. The model was adjusted for WTC exposure (high vs. low), age on 1 March 2020, race (white vs. non-white), sex, former work assignment (EMS vs. firefighter), hypertension, OAD, ILD, smoking history, obesity, and diabetes. All combinations of first order interactions were tested, and none were found to be significant. The final fully adjusted model controlled for confounding variables. In secondary analyses, we also controlled for vaccination status. Statistical significance was indicated if *p*-value was <0.05. Statistical analyses were completed using SAS v9.4 (Cary, NC, USA) and STATA software, version 16.1; College Station, TX, USA; StataCorp, LP.

## 3. Results

The final study population included 8256 participants who had two years of health monitoring followup prior to 1 March 2020 and who had at least one followup exam during the study period. The study characteristics are summarized in Table 1. The majority of participants were male (*n* = 8077, 97.8%) and identified as non-Hispanic white (*n* = 7504; 90.9%). Other less-represented subgroups included Hispanic (*n* = 357; 4.2%), non-Hispanic Black (*n* = 331; 4.0%), Asian (*n* = 30; 0.4%), Native American (*n* = 8, 0.1%) and other race participants (*n* = 26; 0.3%). The mean BMI was 30.3 ±4.7 kg/m^2^. The majority were former firefighters (*n* = 7523; 91.1%), (*n* = 5086; 61.6%) had higher WTC exposure (arrived on 9/11) or (*n* = 3170; 38.4%) had lower WTC exposure (arrived on 9/12 or after). In total, 39.3% were ever smokers (former or current), while (*n* = 5012; 60.7%) never smoked. A total of 41.9% had a physician diagnosis or self-report of hypertension, 51.5% had WTC-related OAD, 1.8% had WTC-related ILD, 5.4% had diabetes, and 46.3% were obese. Overall, 2239 participants were known to have been vaccinated before the end of followup (i.e., before their last healthcare interaction with the WTC Health Program during the period between 1 March 2020 and 1 August 2021). There was no significant difference in vaccination between the WTC exposure groups (27.1% of highly exposed and 27.5% of lesser exposed; χ^2^ *p* = 0.69).

The crude overall cumulative incidence of self-reported COVID-19 infection calculated from 1 March 2020 to 1 August 2021 was 13.1% (130 per 1000) in the overall population and was similar in firefighters and EMS workers (13.2% and 12.0%, respectively). The incidence of severe COVID-19 (requiring hospitalization or death) was low at 0.6% in the overall population (0.6% in firefighters and 1.1% for EMS workers) (Supplementary Table S1). The cumulative incidence was also calculated by level of WTC exposure, as this was a variable of interest. After removing participants with unknown WTC exposure history, 15.1% with higher WTC exposure had COVID-19 versus only 12.0% with lower exposure. To correct for potential bias related to followup time, the incidence rate (IR) and the incidence rate ratio (IRR) by WTC exposure history and work assignment were calculated (Table 2). In this analysis, those with higher WTC exposure were 1.25 more likely to be diagnosed with COVID-19 (IRR = 1.25; 95% CI = 1.10–1.43). There were small non-significant differences between firefighters and EMS workers in their incidence rate (IRR: 1.00; 95% CI = 0.81–1.25). To verify the self-reported data, self-reported hospitalizations and documented hospitalizations were evaluated and had strong agreement (86%). Both participants with and without a COVID-19 diagnosis had the same median number of interactions with the WTC medical monitoring and treatment program (median = 3; Q1–Q3 = 2–5). Additionally, the median number of exams was the same for those with higher and lower WTC exposure (median = 3; Q1–Q3 = 2–5).

**Figure 1:**
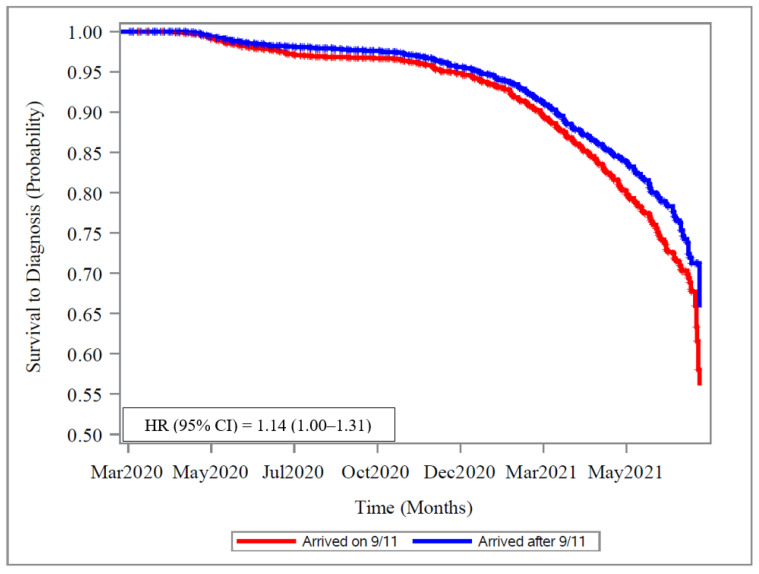
Unadjusted Kaplan–Meier survival curve and adjusted Cox Proportional Hazards regression shows the unadjusted Kaplan–Meier survival curves with the hazard ratio for Arriving on 11 September 2001 vs. Arriving after 11 September 2001 from the adjusted Cox Proportional Hazards regression model in the panel. The model controls for race, sex, age on 1 March 2020, diabetes, interstitial lung disease, obstructive airways disease, obesity, hypertension, and smoking.

Figure 1 displays the unadjusted Kaplan–Meier curves and the results from the adjusted Cox regression model, which included demographic variables, clinical characteristics, and health conditions and assessed which of those were associated with self-reported COVID-19. The outcome was self-diagnosed COVID-19 of all severities as we were underpowered to analyze severe COVID-19 as an outcome. Independent variables included in the model were WTC exposure, age, race, sex, work assignment, smoking history, obesity, hypertension, WTC-associated OAD and ILD, diabetes, and obesity. Higher WTC exposure increased the risk of COVID-19 by 14% when compared to those with lower WTC exposure. Increasing age as a continuous variable decreased risk of COVID-19 by 3% for each year increase in age (HR 0.97; 95% CI 0.96–0.98; *p* < 0.01) (Figure 1, Supplementary Table S2). History of hypertension, OAD, and ILD were not significantly related to a diagnosis of COVID-19, and the main variable of interest, WTC exposure, was not significantly impacted when analysis was completed with or without adjusting for OAD or ILD (data not shown). Similar findings were observed when evaluating the association between WTC exposure and incidence of COVID-19 in the partially adjusted and fully adjusted models, as well as when controlling for vaccine status.

## 4. Discussion

This is one of the first studies to calculate incidence and assess risk factors for COVID-19 in a cohort followed prospectively for decades prior to the pandemic. Furthermore, we sought to evaluate WTC exposure, specifically, as a risk factor for COVID-19. This is one of the few works evaluating an occupational cohort other than that of healthcare workers [12].

Our study found that greater exposure to the hazards at the WTC site (arriving on the day of 9/11) was an independent risk factor for COVID-19 infection even when controlling for other known risk factors or confounders reported in earlier COVID-19 studies such as age, sex, hypertension, obstructive airways disease, diabetes, increased age, and obesity. One possible explanation is that the pollution produced by the WTC collapse increased active inflammation and oxidative stress making one more susceptible to COVID-19, which has been proposed in non-WTC air pollution studies related to COVID-19 [13]. We have demonstrated an increased risk of other diseases thought to be due to chronic inflammation after WTC exposure such as obstructive airways disease, sleep apnea, and cardiovascular disease [14,15,16,17]. Another explanation could be that those with the highest WTC exposure have other characteristics that make them more likely to engage in riskier behaviors in terms of COVID-19 exposure. Further studies are needed to better understand this relationship. We found no difference in the median number of followup exams during the study period for those with higher or lower WTC exposure and no difference between those with or without self-reported COVID-19. There was also no difference in vaccination status for those with higher or lower WTC exposure. We also adjusted for followup time in the calculation of incidence rate and Cox regression modeling, thus minimizing the impact of ascertainment bias.

Unlike other COVID-19 studies, our study did not find obstructive airways disease, obesity, or increased age to be risk factors for COVID-19. Meanwhile, older participants had a decreased risk of having COVID-19. Perhaps obstructive airway disease and obesity were not related to COVID-19, because members of the FDNY retired cohort receive regular medical care and a pension. It is likely they have the resources necessary to manage their health conditions and were able to shelter-in-place more easily than someone without these resources. Additionally, members of this cohort even with obesity and obstructive airways disease, may be healthier overall than those in prior studies, since most studies evaluating risk factors included only hospitalized participants. Regarding age, our findings were similar to those reported in another WTC-exposed cohort of non-FDNY responders in that they also reported that those who were younger were more likely to be diagnosed with COVID-19; however, they reported current employment status in their study and found that those who were employed were more likely to be diagnosed with COVID-19 [6]. Although all in our cohort were retired from FDNY work prior to the pandemic, the youngest individuals may still be in the workforce generally and are also more likely to be socially active even when not working, thereby explaining why younger age was a significant risk for COVID-19. Additionally, approximately 77% of the FDNY WTC cohort are currently retired from FDNY work, which indicates this study is a fair representation of the entire FDNY WTC-exposed cohort. A main strength of this study is that it is one of the few studies that evaluated COVID-19 in a non-hospitalized cohort, though we were limited in that we were underpowered to evaluate severe COVID-19. It is possible that WTC exposure causes a unique combination of physiological abnormalities, which increases an individual’s risk of COVID-19, and perhaps those with this environmental/occupational exposure history should take more strict preventive measures against COVID-19 during future surges. Finally, although this would require medical record information confirming infection, as a limitation of this study was of self-reported COVID-19, future research in this cohort could more accurately explore the incidence and risk factors for post-COVID syndromes given that extensive pre-pandemic data have been classified and recorded.

## 5. Conclusions

We found arriving on the day of 9/11 was an independent risk factor for COVID-19 infection even when controlling for other conditions known to be associated with WTC exposure. Highly exposed recovery workers should be advised of this potential association, so additional precautions may be taken. Because the current study evaluated the period between March 2020 and August 2021, future work should investigate subsequent waves of COVID-19 infection as well as the mechanism behind this potential association.

## Figures and Tables

**Table 1:** Study Cohort Characteristics.

Characteristics	N = 8256
Sex, *n* (%)	
Male	8077 (97.6)
Female	179 (2.2)
Age in years ^1^, mean (SD)	61.2 (8.4)
BMI kg/m^2^ mean (SD)	30.3 (4.7)
Race, *n* (%)	
Non-Hispanic white	7504 (90.9)
Hispanic	357 (4.3)
Non-Hispanic Black	331 (4.0)
Asian	30 (0.4)
Native American	8 (0.1)
Unknown	26 (0.3)
Work Assignment, *n* (%)	
Firefighter	7523 (91.1)
EMS	733 (8.9)
Arrival Time, *n* (%)	
Morning of 11 September	1271 (15.4)
Afternoon of 11 September	3815 (46.2)
12 September	1493 (18.1)
13 September–24 September	1295 (15.7)
After 24 September	181 (2.2)
Unknown/missing	201 (2.4)
Smoking history, *n* (%)	
yes	3244 (39.3)
no	5012 (60.7)
Comorbidities, *n* (%)	
Hypertension	3456 (41.9)
OAD ^2^	4253 (51.5)
Obesity	3819 (46.3)
Diabetes	448 (5.4)
ILD ^2^	148 (1.8)

Abbreviations: BMI, Body Mass Index; OAD, Obstructive Airways Diseases; ILD, Interstitial Lung Diseases; ^1^ Age in years on 1 March 2020; ^2^ World Trade Center certified condition (https://www.cdc.gov/wtc/conditions.html) (accessed on 20 May 2022).

**Table 2:** (**a**) Incidence Rates of COVID-19 by WTC Exposure History. (**b**) Incidence Rates of COVID-19 by Work assignment ^1^.

**(a)**			
**Exposure Group**	**COVID-19 Cases**	**Person-Years**	**Incidence Rate (Per 1000 Person-Years)**
Higher exposure	722	4631.15	155.9
Lower exposure	336	2699.11	124.5
Incidence rate ratio: 1.25 (95% CI 1.10–1.43)			
**(b)**			
**Work Class**	**COVID-19 Cases**	**Person-Years**	**Incidence Rate (Per 1000 Person-Years)**
Firefighter	993	6890.93	144.1
EMS	88	613.21	143.5
Incidence rate ratio: 1.00 (95% CI 0.81–1.25)			

^1^ Removed those with unknown exposure history.

## Data Availability

Reasonable requests for deidentified data will be considered by the investigators.

## Supplementary Materials

The following supporting information can be downloaded at: https://www.mdpi.com/article/10.3390/ijerph19158891/s1, Table S1. (**a**) Cumulative Incidence of COVID-19 by Work Assignment. (**b**) Cumulative Incidence of COVID-19 by Exposure Intensity; Table S2. Cox proportional hazards models *. (**a**) Fully adjusted. (**b**) Partially adjusted. (**c**) Fully adjusted with vaccination status.
