# Supplementary material for: Risk Factors for COVID-19 in a Retired FDNY WTC-Exposed Cohort
Source: Int J Environ Res Public Health. 2022 Jul 22;19(15):8891. doi: 10.3390/ijerph19158891 (PMC9331420; doi:10.3390/ijerph19158891)
Supplement: Supplementary file 1 [file ijerph-19-08891-s001.zip › ijerph-1775475-supplementary.pdf]

Table S1. (**a**) Cumulative Incidence of COVID-19 by Work Assignment. (**b**) Cumulative Incidence of COVID-19 by Exposure Intensity.

(a)

Work Assignment	Severe COVID-19	Not Severe COVID-19	No COVID-19	Total	Cumulative Incidence Severe	Overall Cumulative Incidence
Firefighters	44	949	6530	7523	0.6	13.2
EMS	8	80	645	733	1.1	12.0
Total	52	1029	7175	8256	0.6	13.1

(b)

Exposure Intensity Assignment	Severe COVID-19	Not Severe COVID-19	No COVID-19	Total	Cumulative Incidence Severe	Overall Cumulative Incidence
Higher Exposure	32	690	4364	5086	0.6	14.2
Lower Exposure	20	316	2633	2969	0.6	11.3
Total [1]	52	1006	6997	8055	0.6	13.1

[1] Removed those with unknown exposure history.

Table S2. Cox proportional hazards models *. (**a**) Fully adjusted. (**b**) Partially adjusted. (**c**) Fully adjusted with vaccination status.

(a)

Characteristic	Hazard Ratio	95% CI
WTC Exposure		
High (9/11)	1.14	1.00-1.31
Low (≥9/12)	ref	
Age, years	0.97	0.96-0.98
Sex		
Male	1.36	0.82-2.26
Female	ref	
Race		
White	1.03	0.81-1.32
Non-white	ref	
Smoker		
Ever	0.93	0.82-1.06
Never	ref	
Work assignment		
Firefighter	1.07	0.82-1.40
EMS	ref	
Hypertension	0.89	0.78-1.02
Obesity	1.05	0.92-1.19
OAD	1.10	0.97-1.25
ILD	1.04	0.68-1.58
Diabetes	0.88	0.65-1.18

(**b**)

Characteristic	Hazard Ratio	95% CI
WTC Exposure		
High (9/11)	1.15	1.00-1.31
Low (≥9/12)	Ref	
Age, years	0.964	0.96-0.98
Sex		
Male	1.32	0.80-2.19
Female	ref	
Race		
White	1.05	0.83-1.35

Non-white	ref	
Smoker		
Ever	0.93	0.82-1.06
Never	Ref	
Work assignment		
Firefighter	1.07	0.82-1.40
EMS	ref	

(**c**)

**Characteristic**	**Hazard Ratio**	**95% CI**
WTC Exposure		
High (9/11)	1.14	1.00-1.30
Low (≥9/12)	ref	
Age, years	0.97	0.96-0.98
Sex		
Male	1.34	0.80-2.23
Female	ref	
Race		
White	1.04	0.81-1.33
Non-white	ref	
Smoker		
Ever	0.93	0.82-1.06
Never	ref	
Work assignment		
Firefighter	1.15	0.88-1.51
EMS	ref	
Hypertension	0.86	0.76-0.99
Obesity	1.06	0.94-1.20
OAD	1.16	1.02-1.32
ILD	1.35	0.89-2.07
Diabetes	0.90	0.66-1.21
Vaccination Status		
Vaccinated	0.31	0.27-0.36
No Vaccine	ref	

* N = 224 participants with unknown missing exposure or race data were excluded from the models.
